# Performance of Heat-Insulating Materials Doped with Basalt Fibres for Use in Mines

**DOI:** 10.3390/polym12092057

**Published:** 2020-09-10

**Authors:** Yewei Jiang, Song Xin, Hongyu Li, Long Zhang, Chuanbin Hou, Zhaopeng Zhang, Jianghui Guo

**Affiliations:** 1 College of Safety and Environmental Engineering, Shandong University of Science and Technology, Qingdao 266590, China; jiangyewei1314@163.com (Y.J.); aghlnoz@163.com (L.Z.); chuanbinhou@163.com (C.H.); qdskzzp@126.com (Z.Z.); gjh199609@126.com (J.G.); 2 State Key Laboratory of Mining Disaster Prevention and Control Co-found by Shandong Province and the Ministry of Science and Technology, Shandong University of Science and Technology, Qingdao 266590, China; 3 College of Transportation, Shandong University of Science and Technology, Qingdao 266590, China

**Keywords:** inorganic heat-insulating materials, basalt fibres, orthogonal test, numerical simulation

## Abstract

To solve high-temperature-induced hazards in mines, heat-insulating materials were prepared by utilising basalt fibres and high-strength ceramsite combined with cementing materials. Through orthogonal tests and data analyses, the optimal combination of the heat-insulating materials doped with basalt fibres was determined as A_1_B_1_C_1_, that is, doping with 45% basalt fibres, a length of the basalt fibres of 6 mm, and doping with 20% ceramsite. The performance indices corresponding to the optimal comprehensive combination of the heat-insulating materials doped with basalt fibres included a density of 1200 kg/m^3^, thermal conductivity of 0.151 W/(mK), compressive strength of 9.7 MPa, flexural strength of 3.6 MPa, and a water-seepage depth of 25.4 mm. Numerical simulations verified that the materials presented favourable thermal insulation performance.

## 1. Introduction

Coal is the primary energy source in China, and the coal mining industry promotes the socio-economic development of the nation. With increases in mining depth, the temperature of the rock increases, triggering high-temperature hazards. These hazards affect workers’ physical and mental health, and cause a reduction in their labour output; more seriously, scientific research has shown that high-temperature-induced hazards can also trigger rock collapse and gas explosion accidents, threatening mine safety [1].

At present, high-temperature hazards in mines are ameliorated by applying a mechanical cooling refrigeration system combined with cooling devices set in local areas underground; however, this method relies on the reasonable distribution and application of large-scale refrigeration equipment, which will increase the cost of mine construction. In the trend of pursuing environmental protection and energy saving benefits, it is thus deemed necessary to explore and implement new cooling measures for mines to improve the quality of such cooling systems. In this context, heat-insulating materials provide a new idea for controlling heat hazards in mines.

Heat-insulating materials refer to raw materials characterised by favourable thermal insulation performance, low material density, and suitability for application as spraying and support materials underground, aiming at high-temperature hazards in deep mines [2]. In many deep mines in South Africa, engineers have applied organic heat-insulating materials (e.g., polyethylene foam, polyurethane, and polyurethane foam) to control high-temperature hazards [3]. These materials can be used as heat-insulating materials on account of their low thermal conductivity; however, in the special working environment of high-temperature deep mine roadways, organic materials are easily burned and prone to emitting poisonous and harmful gases which would threaten the safety of workers should a mine fire occur underground. On the other hand, the materials are novel polymer materials, and are expensive, making them uneconomical for large-scale application [4]. In 2017, the All-Russian Scientific Research Institute of Aviation Materials (Moscow, Russia) developed materials with high thermal insulation performance based on heat-insulating materials with fibres. For aircraft, refractory fibres are an effective heat-insulating material [5]. The material, with a low specific thermal conductivity coefficient and low density, is fire resistant and can work for a long time at high temperatures; however, the performance of fibre materials varies with the material volume owing to its lower strength. To address the nonuniformity in the volume of heat-insulating materials with rigid fibres, scientists have conducted multistep heat treatments on rigid fibres. By adjusting the temperature in the treatment process, a heat-insulating material with rigid fibres whose strength and thermal insulation performance both satisfy the industrial requirements was developed [6]. In addition, scholars in some countries (such as Germany and Australia) have explored inorganic heat-insulating materials, including waste slags produced during the industrial processes. However, engineering practice shows that these materials fail to satisfy the basic requirements for use in deep mine roadways [7]. A novel, nonflame-retardant and cheap heat-insulating material proposed by Wang et al. [8] in 2014 has been used in vermiculite plasters in roadways. The interior of the material contains solid phases and significant volumes of gas phases (usually air). The prepared heat-insulating material with a low thermal conductivity coefficient presents good thermal insulation in surrounding rocks in deep mines. However, the material exhibits low permeability owing to the presence of many pores and voids within. As a result, the thermal conductivity coefficient of the material varies in the roadways subject to seepage, and thereby affects its thermal insulation performance. In 2016, Yang et al. [9] prepared specimens from raw materials (e.g., high-strength ceramsite, vitrified microspheres, fly ash, and cement) and used orthogonal tests to determine the thermal conductivity and compressive strength. Afterwards, the optimum proportion of the heat-insulating materials was selected by applying the efficacy coefficient method. The material can satisfy the requirements for service as a support material in terms of some physical and mechanical properties; however, it is necessary to test material indices including flexural strength and permeability to achieve a multifaceted performance. In 2019, Wu et al. [10] conducted related experimental work on the spraying and heat-insulation mechanism of roadways at high ground temperatures and proposed a theory for the design of a heat-resistant composite loop, combining roadway shotcreting with grouting to resist heat transfer from surrounding rocks, together with ventilation to cool by conduction and convection. In this way, the roadways at a high ground temperature are cooled, combining prevention and control measures. By performing tests to measure the strength and thermal conductivity, a heat-insulating material with a density of 1480 kg/m^3^ and thermal conductivity of about 0.15 W/(mK) was developed by taking cement and cement-based vitrified microspheres as raw materials.

Basalt fibres have excellent mechanical properties. Wang et al. [11] studied the effects of different amounts of basalt fibres on the Cl- distribution brought by coral aggregates, splitting tensile strength, and compressive strength in BF-reinforced coral aggregate concrete (CAC). The results indicated that the compressive strength and splitting tensile strength of CAC were enhanced by incorporating basalt fibres, and the addition of 1.5 vol % or 2.0 vol % basalt fibres had a better impact. Cui et al. [12] studied the bonding performance of basalt fibres reinforced cementitious composites through analyses on pull-out tests results. It was concluded that the rupture failure which occurred for dispersed and flexible-type fibres, the pull-out failure which occurred for controlled cluster-type fibres, and the failure modes of alkali-resistant fibres were related to their embedded lengths. As lengths increase, peak pull-out load (N-pmax) increases and then becomes unchanged. In this paper, compressive and flexural tests of insulation materials mixed with basalt fibres are carried out to further verify the role of basalt fibre- reinforced materials in terms of their mechanical properties. In the study of Seghini et al. [13], the fatigue life of a flax-basalt woven-ply hybrid composite was investigated and compared with the behaviour of 100% flax and 100% basalt composites. After the optimisation of the epoxy resin curing cycle, tension–tension fatigue tests were performed on samples with two orientations, i.e., 0/90° and ±45°. The results showed that hybridization was able to produce a positive effect on the fatigue resistance of basalt laminates. Chlup et al. [14] characterised both representative materials with the aim to determine similarities and differences in the fracture processes. The microstructural, elastic, and fracture properties were also examined. The fracture resistance was obtained in two typical directions, i.e., along and across the fibres. Based on this, this paper studies the influence of basalt fibre length on thermal insulation materials, and determines its influence on thermal insulation and mechanical properties.

Basalt fibres feature many excellent physical properties such as low cost, high temperature resistance, stretching resistance, corrosion resistance, and safety during production. They can be degraded into soil parent materials after being abandoned and are, effectively, green industrial materials [15,16,17]. By comparing and analysing three inorganic heat-insulating materials experimentally, Jiang et al. [18] found an optimal test material blended with basalt fibres with a thermal conductivity coefficient of 0.1323 W/(mK) and a compressive strength of 10.98 MPa. The material was able to be used as a heat-insulating material in mines. Heat-insulating materials for mines were prepared by selecting basalt fibres as the main raw materials, and their mechanical properties and thermal insulation performance were experimentally explored to reveal their comprehensive superiority in preparing heat-insulating materials for mines.

## 2. Testing

### 2.1. Test Materials

In light of relevant stipulations in current Chinese standards for cements, 425# Portland cement produced in Qingdao, Shandong Province, China was used to satisfy requirements for sprayed concretes in terms of strength, durability and workability. The fly ash (ceramsite) was prepared by adding a certain amount of fly ash and doping a certain amount of binder and water in the mixing process of raw materials to process the mixture into particles with approximately equal sizes (the diameter of the ceramsite selected in this experiment was 3−5 mm). The ceramsite was prepared through high-temperature calcination, showing a series of advantages, e.g., it was light-weight, of high strength, prepared from abundant raw materials, environmentally-friendly, and cheap. Fly ash ceramsite produced in a construction material factory in Jinan, Shandong Province, was employed in the tests. Basalt fibres were first developed in the Soviet Union in the 20th century. At present, basalt fibres are regarded as the ideal filler for polymer composites and concretes due to their mechanical and thermodynamic performances [19]. The basalt fibres (thermal conductivity was between 0.031–0.038 W/(mK) and the breaking strength was 3500–4300 Mpa) produced by a material factory in Jinan were applied in these tests, and natural medium-grained sands were used as the test aggregates.

### 2.2. Design of the Basic Mixture Proportions

According to the single factor test performed initially, the basic proportions of the heat-insulating materials for mines doped with basalt fibres were set to 1:3.58:0.5 (cement: Aggregate: Water). Some aggregates were replaced by ceramsite.

### 2.3. Orthogonal Test Design

According to the design method of orthogonal tests, three-factor, three-level tests (a total of nine groups of tests) were designed. As shown in Table 1, based on existing research, three factors (i.e., dosage and length of basalt fibres, as well as the dosage of ceramsite) were designed; three levels represented three corresponding dosages of raw materials. The dosages of basalt fibres (percentage of basalt fibres in the total mass of cement) were 45%, 50%, and 55%, respectively; the lengths of the basalt fibres were (separately) 6, 12, and 18 mm, respectively; the dosages of ceramsite were 40%, 45%, and 50%. A total of three factors were considered, and an L_9_ (3^4^) orthogonal table was used in the present experimental design.

### 2.4. Preparation and Maintenance of Specimens

The proportions of raw materials in various groups were designed according to the orthogonal tests, and further raw materials were separately weighed. The high-strength ceramsite, sands, and cements were poured into a stirrer, in which basalt fibres were added after sufficient stirring. To avoid agglomeration of the basalt fibres, manual dispersion was necessary. Afterwards, the stirred concrete specimens were reinforced with basalt fibres and placed into the mould required for different tests; subsequently, the moulds were placed on a horizontal shaking table and vibrated. After preparation, the specimens with different dimensions should be placed apart according to strength class for about 24 h; next, the specimens were demoulded and labelled.

**Figure 1:**
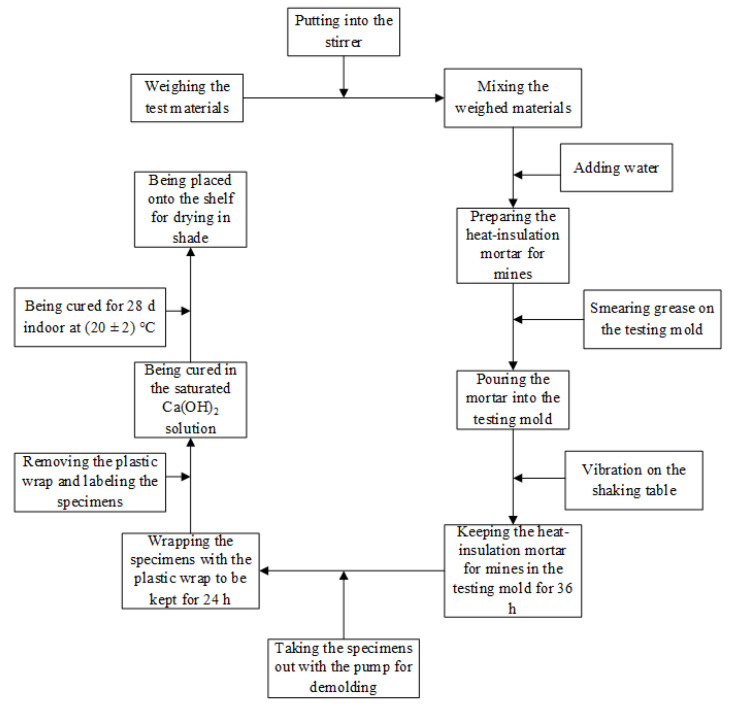
Specimen preparation.

All specimens were placed into a Ca(OH)_2_ saturated solution to be cured. In the curing box, the specimens were not allowed to come into contact with each other, i.e., they were separated by at least 10 mm and always submerged with the solution level over 50 mm above their upper surfaces. The specimens were cured for 28 days indoors at a temperature of (20 ± 2) °C to avoid exposure to sunlight. After 28 days, the specimens were removed from the curing tank and air-dried in shade under indoor conditions at constant temperature (Figure 1).

### 2.5. Test Instruments and Methods

#### 2.5.1. Compressive Strength Testing

The compressive strengths of nine groups of specimens (nonstandard specimens measuring 100 mm × 100 mm × 100 mm) were tested. To improve the accuracy and reliability of the test data, three replicates were tested. An AGX-250 electronic universal testing machine (Shimadzu Corporation, Kyoto, Japan) was used to measure the compressive strength.

#### 2.5.2. Flexural Strength Testing

According to the relevant stipulations of the standard on the test method of the mechanical properties of sprayed concretes and the test for flexural strength, the flexural strength was measured by using beam-type standard specimens measuring 150 mm × 150 mm × 550 mm. Three such specimens were designed as a group, and a total of nine groups were conducted. A YDW-10 microcomputer-controlled electric flexural testing machine (Xingaoweiye Scientific, Tianjin, China) was used.

The specimens should be used timeously after curing. The specimens were wiped and cleaned, and then installed according to “Standard for Test Methods of Mechanical Properties of Ordinary Concrete” (GB/T 50081—2002), with the deviation of the installation dimensions being no greater than 1 mm. The flank of a formed specimen was supposed to act as the load-bearing surface thereof. The pedestal and the support surface were steadily and uniformly brought into contact with the cylinder before loading at 0.02 to 0.05 MPa/s. When a specimen was almost damaged, it was necessary to stop adjusting the accelerator of the testing machine until the specimen was damaged; afterwards, the failure load was recorded. The reading on the testing machine under the failure load and the fracture position of the lower edge of the specimen were recorded.

#### 2.5.3. Permeability Testing

A HP-4.0 automatic pressure regulating concrete permeability meter (Jingrui Instrument, Cangzhou, China), was used. The specimen measured 175 mm × 185 mm × 150 mm, and three replicates were tested.

The cured specimens were taken out one day before testing. The surface of the specimens was dried in the sun and the testing mould was heated to 40 °C. Moreover, after heating the sealing wax until it was completely melted, the specimens were rolled through a circle in the melted wax (without getting wax on the two end faces of the specimens, which could be protected by pasting newspapers thereon). The specimens coated with wax were pressed into the testing mould preheated by the drying oven. After being cooled slightly, the pressure could be removed and the specimens together with the testing mould were nested on the permeability meter. The nut of the watering gun was screwed off and various valves were opened; subsequently, a funnel was connected to the water-injection nozzle to inject water into the water tank (the chassis of the testing mould was also filled with water to displace air in the pipeline system). After they were filled with water, six valves feeding water to the testing mould were closed. The packed testing mould with its specimens was fixed onto the instrument. At first, the small valve was opened and the No. 0 control valve was closed. Until the linear water flow was generated from the small water nozzle, the six control valves were opened and the small water valve was closed. The initial water pressure was set as 0.1 MPa, and then water pressure was increased by 0.1 MPa at intervals of 8 h. The water seepage of the upper end face of the specimens was observed until the test was ceased when the end faces of three of six specimens showed seepage, and the water pressure at that time was recorded. The water-seepage height can be measured by fracturing the water-seeping specimens at the middle using the fracturing equipment. The impermeability is calculated as follows:

(1) S=10 H−1

where *S* and *H* refer to the degree of impermeability and water seepage pressure (MPa), respectively.

#### 2.5.4. Thermal Conductivity Testing

A DRPL-1 thermal conductivity coefficient instrument (Xiangtan Instrument, Hunan, China), was applied in the test. The measuring range of instrument thermal conductivity was 0.015–5 W/(mK), and heat-insulating materials measuring 100 mm × 100 mm × 100 mm were tested using the steady-state plate method. The instrument was divided into the upper (hot) and lower (cold) surfaces. After setting the temperature, the heat was transferred to the cold surface through the specimens; afterwards, according to the thickness (100 mm) and the heat transfer area (10,000 mm^2^) of the specimens, the thermal conductivity of the heat-insulating materials was calculated.

## 3. Analysis of Test Data

### 3.1. Analysis of the Apparent Density

Apparent density refers to the mass of material particles of unit apparent volume (including material solid volume and volume of closed pores) in a natural state, which is calculated as follows:

(2) $$\rho^{\prime }=\;\frac{m}{v^{\prime }}$$

where $\rho^{\prime }$, m and $v^{\prime }$ denote the apparent density (kg/m^3^), the mass (kg), and volume (m^3^) of materials, respectively.

It can be seen from the statistical results in Table 2 (A-dosage of basalt fibres, B-length of basalt fibres, C-dosage of ceramsite) that the apparent density of the specimens in the test groups ranged from 1170 to 1480 kg/m^3^. Compared with the sprayed concrete (with an apparent density of 2100 to 2600 kg/m^3^) commonly used in underground coal mines, the apparent density of the heat-insulating materials was lower, showing the advantage of being light.

### 3.2. Data Analysis: Compressive Strength

An L_9_ (3^4^) orthogonal table was employed and three tests were performed at each factor level. K_i_ represents the sum of the three test results at the *i*th level; on this basis, the mean k_i_ was attained by dividing K_i_ by the number of levels. After preliminary processing of the data, the result is displayed in Table 3.

**Figure 2:**
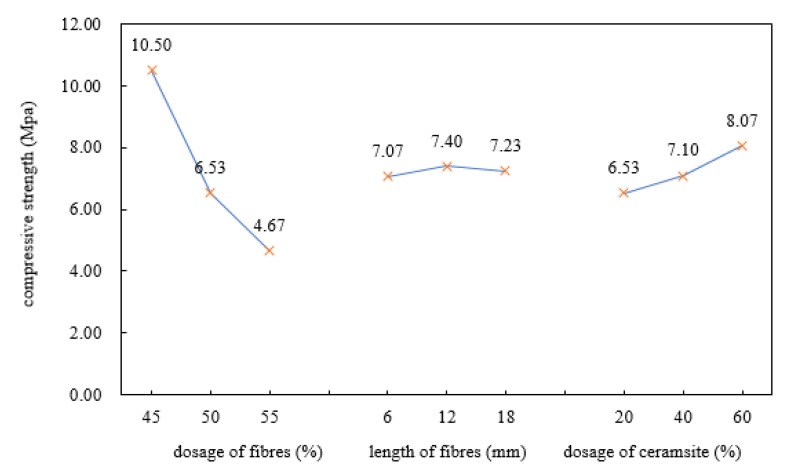
Changes in compressive strength.

To explore the trend in the compressive strength, the range results during the orthogonal tests were plotted and analysed, as shown in Figure 2.

According to Table 3 and Figure 2, it can be seen that in terms of the importance, the compressive strength was significantly affected by the dosage of basalt fibres, i.e., the larger the dosage, the lower the compressive strength. The dosage of ceramsite was negatively related to the compressive strength, and the length of fibres had no obvious influence on the compressive strength.

The three factors influencing the heat-insulating materials were (in descending order): (A) dosage of basalt fibres, (C) dosage of ceramsite and (B) length of basalt fibres. As for the strength of the materials, the higher the compressive strength, the better the mechanical properties thereof. Therefore, we should select those factors with the maximum K value in various columns.

Owing to K_1_ > K_2_ > K_3_ in the column for factor A; K_2_ > K_3_ > K_1_ in the column for factor B, and K_3_ > K_2_ > K_1_ in the column for factor C, the optimal scheme combination was A_1_ B_2_ C_3_ in terms of the compressive strength, that is, the optimal proportion for the compressive strength of the heat-insulating materials was listed as follows: dosage of basalt fibres of 45%, length of basalt fibres of 12 mm, and dosage of ceramsite of 60%.

### 3.3. Data Analysis: Flexural Strength

The measured flexural strengths are listed in Table 4.

**Figure 3:**
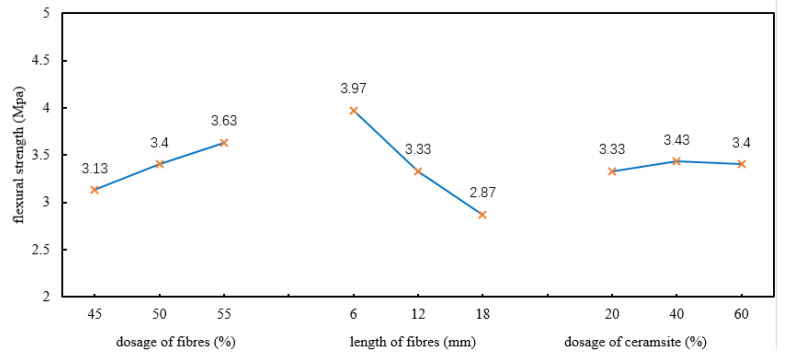
Trend in the flexural strength.

To investigate the trend in the flexural strength, the range results during the orthogonal tests were drawn and analysed (Figure 3).

As shown in Table 4 and Figure 3, in terms of the importance, the length of fibres has a significant effect on the flexural strength, showing a negative correlation. While the increase of the dosage of fibres can increase the flexural strength of the material, the dosage of ceramsite has no obvious effect on the flexural strength.

The three factors influencing the flexural strength of the heat-insulating materials were (in descending order): (B) length of basalt fibres, (A) dosage of basalt fibres, and (C) dosage of ceramsite. For the flexural strength, the higher the flexural strength, the better the mechanical properties of the developed materials. Thus, it was necessary to select the factors with the maximum K value in each column.

Due to K_3_ > K_2_ > K_1_ in the column for factor A; K_2_ > K_3_ > K_1_ in the column for factor B, and K_1_ > K_2_ > K_3_ in the column for factor C, A_3_ B_1_ C_2_ were regarded as the optimal scheme combination in terms of the flexural strength, that is, the optimal proportion for the flexural strength of the heat-insulating materials was described as follows: doping with 55% basalt fibres, using basalt fibres with a length of 6 mm, and doping with 40% ceramsite.

### 3.4. Data Analysis: Impermeability

The impermeability test was conducted on the basis of the water-seepage height. The test data are presented in Table 5.

**Figure 4:**
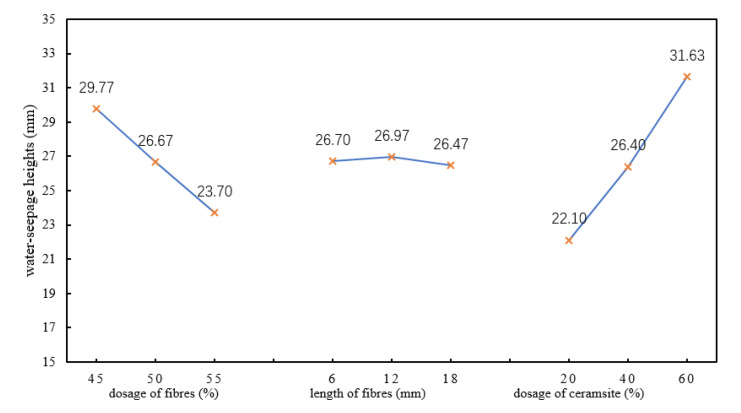
Trend in measured water-seepage height.

To investigate the trend in the impermeability, the range results found during orthogonal tests were plotted and analysed, as shown in Figure 4.

According to Table 5 and Figure 4, an increase in the dosage of ceramsite, as the aggregate in the material, would increase the void ratio in the finished concrete. Although this meant that cementing materials were combined with the aggregates to form a skeletal void structure to some extent, contributing to certain enhancement of the strength of the materials, it significantly affected the permeability of the materials. Due to the growth of the dosage of basalt fibres, a crossed network structure was formed in the heat-insulating materials, which blocked the pore water and prevented capillary water flow. Therefore, increasing the dosage of the basalt fibres in the mixture within a certain range was conducive to improving the permeability of the material. It can be seen that in terms of the importance, the three factors influencing the impermeability of the heat-insulating materials were (in descending order) as: (C) dosage of ceramsite, (A) dosage of basalt fibres, and (B) length of basalt fibres. It was better to have a lower permeability of materials. Thus, it was necessary to select the factors with the minimum K value in each column.

Due to K_3_ < K_2_ < K_1_ in the column for factor A; K_3_ < K_1_ < K_2_ in the column for factor B, and K_1_ < K_2_ < K_3_ in the column for factor C, the optimal scheme combination was A_3_B_3_C_1_ in terms of the impermeability, that is, the optimal proportion for the impermeability of the heat-insulating materials was as follows: doping with 55% basalt fibres, using basalt fibres with a length of 18 mm, and doping with 20% ceramsite.

### 3.5. Data Analysis: Thermal Conductivity

The measured thermal conductivity is listed in Table 6.

**Figure 5:**
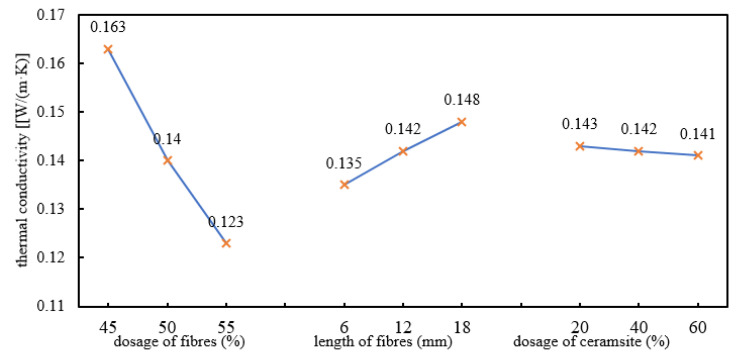
Trend in the thermal conductivity.

To investigate the trend in the thermal conductivity, the range results during the orthogonal tests were plotted and analysed, as shown in Figure 5.

As shown in Table 6, the ranges among three columns for factors A, B, and C were different, implying that the change of the three factor levels exhibited different influences on the thermal conductivity. According to the importance, the three factors are (in descending order): (A) (dosage of basalt fibres), (B) (length of basalt fibres), and (C) (dosage of ceramsite).

According to Figure 5, it can be found that the thermal conductivity varied significantly with the change in the dosage of basalt fibres. There was a negative correlation between the two, and the dosage presented a significant influence on the index. From the perspective of the length of basalt fibres, the thermal conductivity was low when the length of the selected materials was 6 mm. As the length of the basalt fibres varied from 6 mm to 18 mm, the thermal conductivity coefficient gradually rose. In the stirring process, it was difficult to mix the basalt fibres with the other materials after their length exceeded a certain value, and therefore, the mixture failed to be sufficiently stirred. Through analysis according to the change of the dosage of ceramsite in the third column, it was found that the thermal conductivity coefficient was marginally changed after ceramsite was added into the developed materials. Thus, ceramsite exerted a slight influence on the thermal conductivity of the heat-insulating materials.

According to the test results, due to K_1_ > K_2_ > K_3_ in the column for factor A; K_3_ > K_2_ > K_1_ in the column for factor B, and K_1_ > K_2_ > K_3_ in the column for factor C, the optimal scheme combination for the thermal conductivity was A_3_B_1_C_3_, that is, the optimal proportion of the heat-insulating materials was as follows: the optimal dosage of the basalt fibres was 55%, the optimal length of the basalt fibres was 6 mm, and the dosage of ceramsite was 60%.

### 3.6. Comprehensive Analysis: The Efficacy Coefficient Method

By measuring the compressive strength, flexural strength, degree of impermeability, and thermal conductivity of these materials, the optimal scheme combinations in terms of the compressive strength, flexural strength, degree of impermeability, and thermal conductivity were A_1_B_2_C_3_, A_3_B_1_C_2_, A_3_B_3_C_1_, and A_3_B_1_C_3_, respectively. To determine the optimal mixture design, these were also analysed by applying the efficacy coefficient method.

The efficacy coefficient method is a method for solving the multiobjective decision problem [20]. At first, the number *n* of the final indices for data analysis in the design test was determined, with the total of five indices (*n* = 5) in the test: apparent density, compressive strength, flexural strength, degree of impermeability, and thermal conductivity coefficient of materials. Among the nine groups of test data for a single index, the efficacy coefficient of an optimal combination for a performance index was set to 1, that is, *d_i_* = 1. The efficacy coefficient of any other test combination was equal to the ratio of test data of the combination to those of the optimal combination. The total efficacy of each test group was calculated using Equation (3) and the proportion of the test group with the maximum total efficacy coefficient was considered to be the optimal mixture design.

(3) $$d=\;\sqrt[{n}]{d_{1}d_{2}\ldots d_{n}}$$

As shown in Table 7, the total efficacy coefficient (0.83) of the first group of data was found to be the largest after processing the nine groups of data. Therefore, the optimal combination was A_1_B_1_C_1_, that is, doping with 45% basalt fibres, using basalt fibres with a length of 6 mm, and doping with 20% ceramsite. The corresponding performance indices of this combination of the heat-insulating materials were listed as follows: an apparent density of 1200 kg/m^3^, a thermal conductivity of 0.151 W/(mK), a compressive strength of 9.7 MPa, a flexural strength of 3.6 MPa, and a water-seepage height of 25.4 mm, respectively.

## 4. Simulation of the Thermal Insulation

The related simulations of the heat-insulating materials doped with basalt fibres were conducted by applying COMSOL Multiphysics software [21]. By comparing the simulation effects of the heat-insulating materials and common concretes with a same thickness, the thermal insulation effect of the heat-insulating materials in high-temperature mine roadways was verified.

A model was established based on the practical parameters of an auxiliary uphill haulage-way of a certain mine at the depth of 900 m underground. The specific parameters are listed in Table 8.

**Figure 6:**
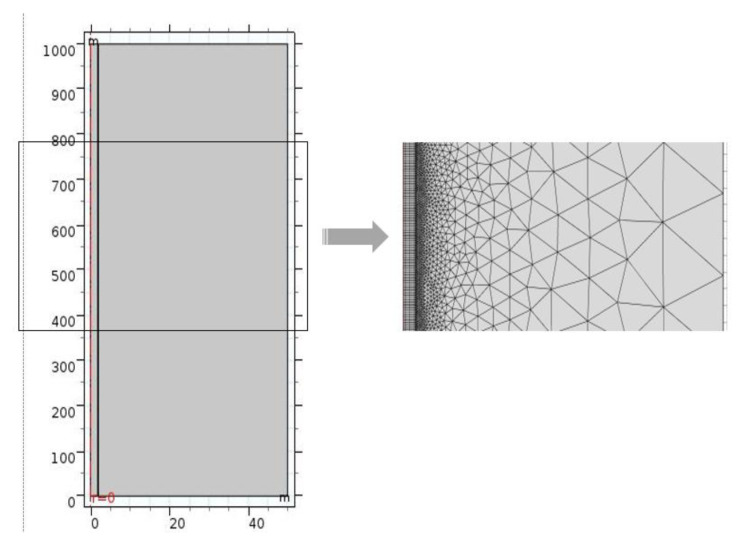
The physical model and partial grid partition.

A physical simulation model was established based on the aforementioned scheme design and parameters; thereafter, grid partition was applied to the established physical model for the roadway. Not every domain is shown due to a large aspect ratio, and therefore, the vertical coordinate (length direction of the roadway) is lowered by a factor of 10 to show the geometric model (Figure 6). In the process of grid partition, it is necessary to refine the grids of the roadway, as they are the most important part of the mesh. Corresponding grids for the roadway are further refined; afterwards, the grid quality is examined to ensure that all elements satisfy the computational demand imposed during subsequent simulation.

**Figure 7:**
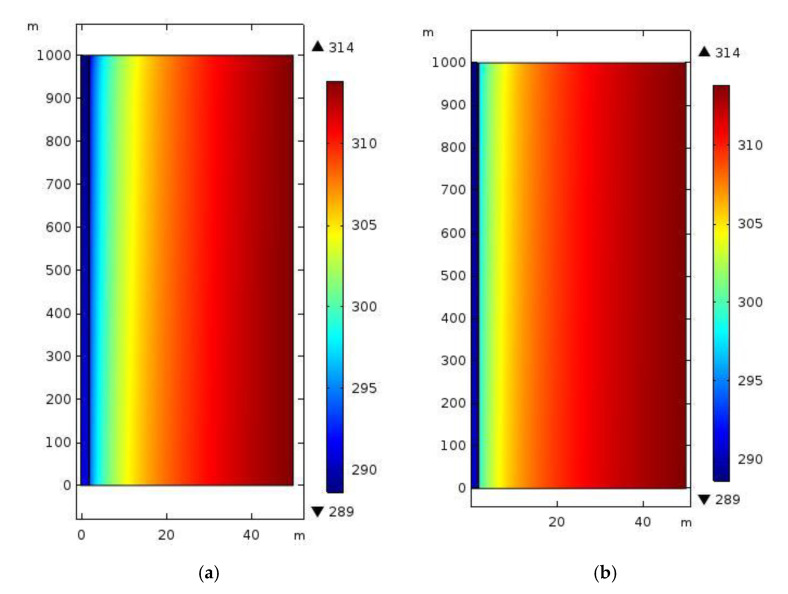
Comparison of the effects of thermal insulation of support materials (transverse section): (**a**) Nephogram of the temperature field of the roadway supported by using common sprayed concrete; (**b**) Nephogram of the temperature field of the roadway supported by using the new heat-insulating materials with basalt fibres.

A numerical simulation was performed on the roadway by separately taking the common sprayed concretes and sprayed heat-insulating materials A_1_B_1_C_1_ as supporting materials in the ventilation area. The nephograms of the temperature field of the two materials are displayed in Figure 7.

For common concretes and heat-insulating materials A_1_B_1_C_1_ (when sprayed to the same thickness), the external boundary conditions were consistent and the temperature of the airflows at the air inlet was the same. In Figure 7a, the temperature of surrounding rocks close to the profile of the roadway changed: The thermal conductivity of the common sprayed concretes used as a support material was relatively high; the heat dissipation in surrounding rocks would exert a larger influence on the airflow temperature when cold airflows constantly went through the roadway, and more cold energy was used to balance the heat dispersed from the surrounding rocks. Therefore, more heat was dispersed from surrounding rocks to the roadway through the common sprayed concrete (support materials) per unit time, which led to an increased airflow temperature. Due to the gradual increase of the airflow temperature, the regulating effect of airflows on surrounding rocks along the direction of the length of the roadway was gradually weakened and their zone of influence also shrank. In Figure 7b, the characteristic of the nephogram formed due to the heat exchange between airflows and surrounding rocks when air flowed through the roadway was less significant than that in Figure 7a. The main reason for this was that the thermal conductivity (0.151 W/(mK)) of the heat-insulating materials for mines doped with basalt fibres was relatively low. This impaired the dissipation of heat from surrounding rocks to the roadway, thus improving the efficiency of utilisation of the cold energy carried by the airflow, so that the heat-insulating materials exerted a significant thermal-insulating effect.

**Figure 8:**
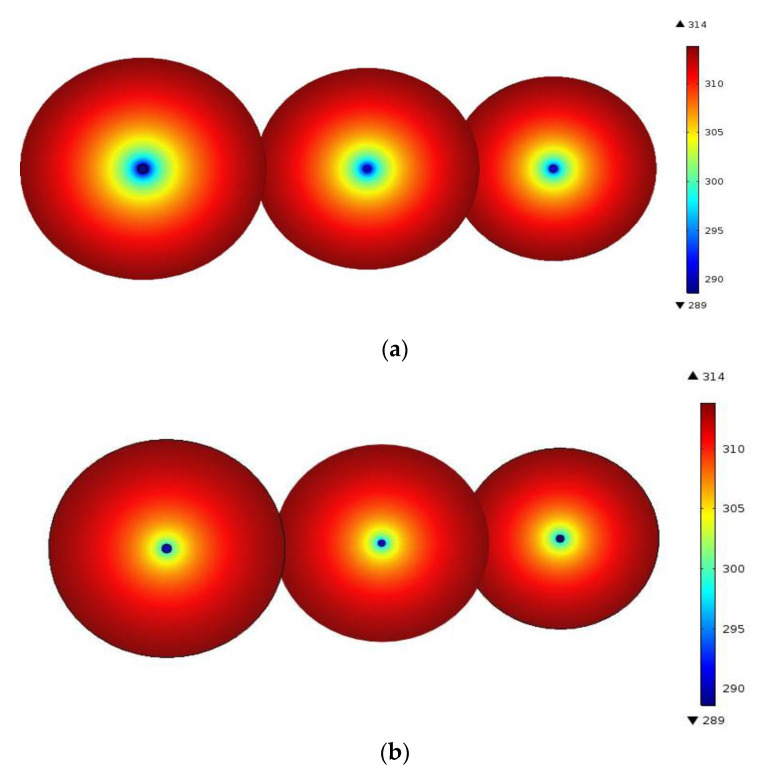
Comparison of effects of thermal insulation of support materials (longitudinal section): (**a**) Nephogram of the temperature field of the roadway supported using common sprayed concrete; (**b**) Nephogram of the temperature field of the roadway supported by using the new heat-insulating materials with basalt fibres.

It can be seen from Figure 8a that the area of the circles of the same colour gradually shrank through analysis according to the temperature distribution of three transverse sections at the air inlet, middle part, and air outlet. This indicated that the temperature difference between airflows and surrounding rocks of the roadway gradually decreased as the surrounding rocks constantly transferred heat to the interior of the roadway; therefore, the effect of thermal insulation of the airflows on surrounding rocks was gradually diminished. In Figure 8b, although the change was relatively insignificant, it was evident that there was a similar trend throughout.

Through comparison, it can be seen that as support materials, these new heat-insulating materials present favourable thermal insulation at the same sprayed thickness.

**Figure 9:**
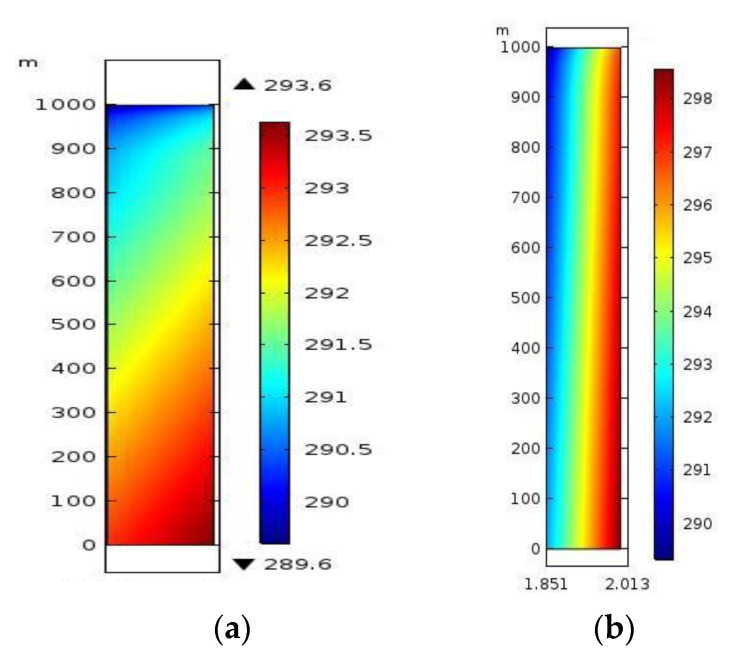
Nephograms of the internal temperature field of support materials: (**a**) Nephogram of the internal temperature field of the common sprayed concretes; (**b**) Nephogram of the internal temperature field of the heat-insulating materials doped with basalt fibres.

The temperature change in the materials can be analysed. Figure 9 shows the nephograms of the change of the internal temperature fields of the common sprayed concrete and heat-insulating material A_1_B_1_C_1_ containing basalt fibres.

**Figure 10:**
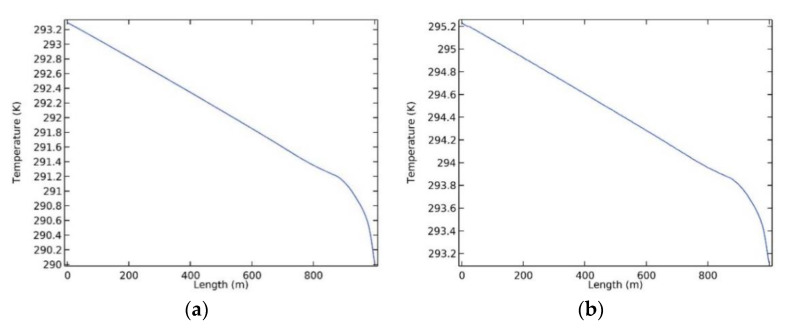
Changes of the internal temperatures of support materials along their centreline: (**a**) Change of the internal temperature of the common sprayed concrete; (**b**) Change of the internal temperature of the heat-insulating materials doped with basalt fibres.

As shown in Figure 9, the temperature distribution at different positions of the materials was irregular due to being influenced by the airflow temperature at the air inlet; however, the internal temperature of materials along the airflow direction from the air inlet (1000 m) to the air outlet (0 m) gradually increased. In addition, owing to the temperature of surrounding rocks being relatively high and that of the airflows being relatively low, heat exchange occurred between the surrounding rocks and airflows in the roadway through the materials. This caused that the temperature of the heat-insulating materials to fall towards the interior of the roadway on the transverse section of the materials. Compared with the heat-insulating materials A_1_B_1_C_1_ with basalt fibres, the common sprayed concrete had a higher thermal conductivity and its heat exchange with the airflows was greater. Thus, the temperature increased rapidly along the air flow. As shown in Figure 9, the thermal insulation performance of the heat-insulating materials A_1_B_1_C_1_ was superior to that of the common sprayed concrete. It can be also seen from Figure 10 that the internal temperature of the common sprayed concrete varied between 290 K to 293.2 K, and that of the heat-insulating materials varied between 293.2 K and 295.2 K. These ranges also reflected (to some extent) that the barrier performance of the heat-insulating materials for heat exchange was better than that of the common sprayed concrete.

**Figure 11:**
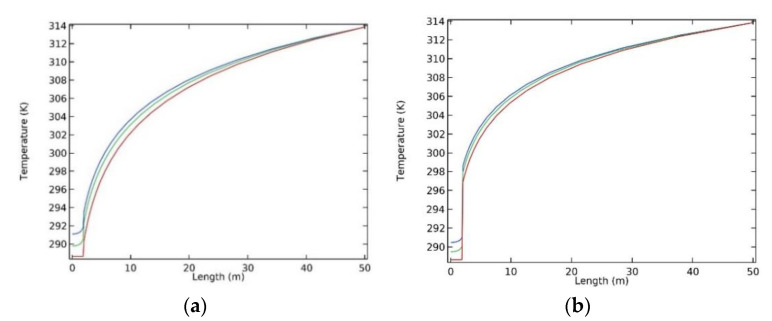
Statistics pertaining to the temperature distributions in the materials: (**a**) The temperature of the common sprayed concrete; (**b**) The temperature of the heat-insulating materials doped with basalt fibres.

A straight line perpendicular to the centreline of the roadway was separately drawn at the inlet (1000 m), middle (500 m), and outlet (0 m) of the model. The temperature data were subjected to statistical analysis along the straight lines from the end of the lines close to the wall surface of the roadway. The statistical results of the temperatures of the common sprayed concrete and heat-insulating materials doped with basalt fibres are displayed in Figure 11.

It can be seen from Figure 11 that the temperatures at the air inlet and outlet of the roadway were separately the lowest and highest. The temperature was about 314 K at the position with a horizontal coordinate of 50 m, that is, the external surface of the model. By taking the temperature at the air outlet as an example, as shown in Figure 11a, the heat from surrounding rocks was gradually transferred to the interior of the roadway along the direction from 50→0 (horizontal coordinate, m), and the whole curve smoothly declined, to about 291.7 K at the wall surface (2 m) of the roadway. The reduction in temperature slowed under the influence of the sprayed concrete. In Figure 11b, the heat from surrounding rocks was also transferred to the interior of the roadway along the direction from 50→0; however, when the heat was transferred to the wall surface (2 m) of the roadway, the temperature of surrounding rocks decreased linearly to about 290.2 K due to the presence of the heat-insulating materials doped with basalt fibres, showing a temperature difference of 1.2 K compared to the common sprayed concrete at the air outlet. This suggested that the heat-insulating materials exhibited satisfactory thermal insulation.

## 5. Conclusions

(1) By performing orthogonal tests and data analyses, the optimal proportion of materials for a single index was separately analysed. The apparent densities of the nine groups of specimens were between 1170 and 1480 kg/m^3^, indicating that the heat-insulating materials doped with basalt fibres were light; the optimal scheme combinations for the compressive strength, degree of impermeability, and flexural strength were A_1_B_2_C_3_, A_3_B_3_C_1_, and A_3_B_1_C_2_, respectively. This provides a basis for further multi-index comprehensive analyses of heat-insulating materials doped with basalt fibres.
(2) The thermal conductivity of the materials gradually decreased with increasing proportion of basalt fibres therein, the dosage of which greatly influenced the indices assessed here. Basalt fibres with three different specifications were selected for testing. The thermal conductivity was low when the length of the selected materials was 6 mm. The test result showed that shorter basalt fibres were more beneficial in improving the thermal insulation performance of the materials. By analysing the change of the dosage of ceramsite, it can be seen that the thermal conductivity changed slightly with the increase in the dosage of ceramsite.
(3) By comparing the simulations of the heat-insulating materials doped with basalt fibres and common sprayed concrete in terms of their relative cooling effects, it can be found that the difference of the internal temperatures was 3 K and the temperature difference at the air outlet was 1.5 K. This verifies the effect of thermal insulation of materials doped with basalt fibres.

## Figures and Tables

**Table 1:** Factors and levels of the orthogonal tests.

Level	Dosage of Basalt Fibres A (%)	Length of Basalt Fibres B (mm)	Dosage of Ceramsite C (%)
1	45	6	20
2	50	12	40
3	55	18	60

**Table 2:** Statistics pertaining to the calculated apparent densities.

Test Number	Level Combination	Experimental Variables			Apparent Density (kg/m^3^)
		A (%)	B (mm)	C (%)	
1	A_1_B_1_C_1_	45	6	20	1200
2	A_1_B_2_C_2_	45	12	40	1420
3	A_1_B_3_C_3_	45	18	60	1480
4	A_2_B_2_C_3_	50	12	60	1350
5	A_2_B_3_C_1_	50	18	20	1260
6	A_2_B_1_C_2_	50	6	40	1310
7	A_3_B_3_C_2_	55	18	40	1230
8	A_3_B_1_C_3_	55	6	60	1280
9	A_3_B_2_C_1_	55	12	20	1170

**Table 3:** Statistics pertaining to the measured compressive strengths.

Test Number	Level Combination	Experimental Variables			Compressive Strength (MPa)
		A (%)	B (mm)	C (%)	
1	A_1_B_1_C_1_	45	6	20	9.7
2	A_1_B_2_C_2_	45	12	40	10.3
3	A_1_B_3_C_3_	45	18	60	11.5
4	A_2_B_2_C_3_	50	12	60	7.6
5	A_2_B_3_C_1_	50	18	20	5.6
6	A_2_B_1_C_2_	50	6	40	6.4
7	A_3_B_3_C_2_	55	18	40	4.6
8	A_3_B_1_C_3_	55	6	60	5.1
9	A_3_B_2_C_1_	55	12	20	4.3
K_1_		31.50	21.20	19.60	
K_2_		19.60	22.20	21.30	
K_3_		14.00	21.70	24.20	
k_1_		10.50	7.07	6.53	
k_2_		6.53	7.40	7.10	
k_3_		4.67	7.23	8.07	
range		17.5	1	4.6	
preferred plan		A_1_B_2_C_3_			

**Table 4:** Statistics pertaining to the measured flexural strengths.

Test Number	Level Combination	Experimental Variables			Flexural Strength (MPa)
		A (%)	B (mm)	C (%)	
1	A_1_B_1_C_1_	45	6	20	3.6
2	A_1_B_2_C_2_	45	12	40	3.2
3	A_1_B_3_C_3_	45	18	60	2.6
4	A_2_B_2_C_3_	50	12	60	3.3
5	A_2_B_3_C_1_	50	18	20	2.9
6	A_2_B_1_C_2_	50	6	40	4.0
7	A_3_B_3_C_2_	55	18	40	3.1
8	A_3_B_1_C_3_	55	6	60	4.3
9	A_3_B_2_C_1_	55	12	20	3.5
K_1_		9.4	11.9	10	
K_2_		10.2	10	10.3	
K_3_		10.9	8.6	10.2	
k_1_		3.13	3.97	3.33	
k_2_		3.40	3.33	3.43	
k_3_		3.63	2.87	3.40	
range		1.5	3.3	0.3	
preferred plan		A_3_B_1_C_2_			

**Table 5:** Statistics pertaining to the measured water-seepage heights.

Test Number	Level Combination	Experimental Variables			Water Seepage Height (mm)
		A (%)	B (mm)	C (%)	
1	A_1_B_1_C_1_	45	6	20	25.4
2	A_1_B_2_C_2_	45	12	40	29.6
3	A_1_B_3_C_3_	45	18	60	34.3
4	A_2_B_2_C_3_	50	12	60	32.1
5	A_2_B_3_C_1_	50	18	20	21.7
6	A_2_B_1_C_2_	50	6	40	26.2
7	A_3_B_3_C_2_	55	18	40	23.4
8	A_3_B_1_C_3_	55	6	60	28.5
9	A_3_B_2_C_1_	55	12	20	19.2
K1		89.30	80.10	66.30	
K2		80.00	80.90	79.20	
K3		71.10	79.40	94.90	
k1		29.77	26.70	22.10	
k2		26.67	26.97	26.40	
k3		23.70	26.47	31.63	
Range		18.20	1.50	28.60	
Preferred plan		A_3_B_3_C_1_			

**Table 6:** Statistics pertaining to the thermal conductivity.

Test Number	Level Combination	Experimental Variables			Thermal Conductivity (W/(mK))
		A (%)	B (mm)	C (%)	
1	A_1_B_1_C_1_	45	6	20	0.151
2	A_1_B_2_C_2_	45	12	40	0.163
3	A_1_B_3_C_3_	45	18	60	0.175
4	A_2_B_2_C_3_	50	12	60	0.133
5	A_2_B_3_C_1_	50	18	20	0.147
6	A_2_B_1_C_2_	50	6	40	0.140
7	A_3_B_3_C_2_	55	18	40	0.123
8	A_3_B_1_C_3_	55	6	60	0.116
9	A_3_B_2_C_1_	55	12	20	0.131
K_1_		0.489	0.407	0.429	
K_2_		0.420	0.427	0.426	
K_3_		0.370	0.445	0.424	
k_1_		0.163	0.135	0.143	
k_2_		0.140	0.142	0.142	
k_3_		0.123	0.148	0.141	
Range		0.119	0.038	0.005	
preferred plan	A_3_B_1_C_3_				

**Table 7:** Analysis of the efficacy coefficient.

Test Number	Efficacy Coefficient					Final Efficacy Coefficient
	Apparent Density (kg/m^3^)	Compressive Strength (MPa)	Flexural Strength (MPa)	Water Seepage Height (mm)	Thermal Conductivity (W/(mK))	
1	0.98	0.84	0.84	0.76	0.77	0.83
2	0.82	0.90	0.74	0.65	0.71	0.76
3	0.79	1.00	0.60	0.56	0.66	0.71
4	0.87	0.66	0.77	0.60	0.87	0.74
5	0.93	0.49	0.67	0.88	0.79	0.73
6	0.89	0.56	0.93	0.73	0.83	0.78
7	0.95	0.40	0.72	0.82	0.94	0.73
8	0.91	0.44	1.00	0.67	1.00	0.77
9	1.00	0.37	0.81	1.00	0.89	0.77

**Table 8:** Parameters of the simulated roadway.

Part Name	Parameter Name	Model Parameters
Roadway	Shape	Cylinder
	Length/m	1000
	Outer diameter/m + temperature/K	50 + 313.85
	Inner diameter/m + temperature/K	2 + 307.2
Air current	Speed/(m/s)	2.5
	Density/(kg/m^3^)	1.2
Surrounding rock	Thermal conductivity/(W/(mK))	2.1
	Thermal conductivity/(m^2^/s)	0.843 × 10^−5^
Shotcrete	Support thickness/m	0.15
Other	Convection heat transfer coefficient /(W/(m^2^K))	24.35
Ordinary concrete shotcrete material	Density/(kg/m^3^)	2400
	Thermal conductivity/(W/(mK))	1.5
A_1_B_1_C_1_ insulation materials	Density/(kg/m^3^)	1200
	Thermal conductivity/(W/(mK))	0.151
